# Melorheostosis: A Pediatric Case of a Rare Association With Carpal Tunnel Syndrome

**DOI:** 10.7759/cureus.45773

**Published:** 2023-09-22

**Authors:** Yasmina Aboufirass, Afarine Madani

**Affiliations:** 1 Radiodiagnosis, Hôpital Erasme, Brussels, BEL; 2 Radiology, Hôpital Erasme, Brussels, BEL

**Keywords:** surgery, benign, rare, carpal tunnel syndrome, melorheostose, pediatric

## Abstract

Melorheostosis is a rare chronic disease commonly affecting long bones of the lower extremity with the typical imaging feature of hyperostosis “candle wax pattern.” Typically associated with pain, deformities, stiffness, and joint movement restriction (due to contracture and fibrosis), it may also be asymptomatic. Melorheostosis is considered a benign disease but can be extremely debilitating, especially in a pediatric context where progression can be faster than in adults. An even rarer occurrence seems to be its association with nerve impingement. In this paper, we present the case of an 8-year-old girl with a known condition of melorheostosis of the upper limbs who developed bilateral carpal syndrome. To our knowledge, very few cases of the sort have been described, and even less in a pediatric context.

## Introduction

Melorheostosis is a rare condition causing sporadic sclerosing mesodermal dysplasia affecting bones and soft tissues with an incidence of 0.9 cases per million [1]. Any age group and gender may be affected. Dysplasia occurs in early childhood while adults present contracture and pain. In the former group, 40-50% of cases are evident by the age of 20 [2]. Although melorheostosis is considered a benign disease, pain, and deformity can be a cause of functional morbidity especially in children where progression can be faster than in adults [3]. Commonly affecting long bones of the lower extremity (with the typical imaging characteristic feature of hyperostosis “candle wax pattern”), the involvement of small bones of the hand is uncommon [1]. Even more uncommon is its association with carpal tunnel syndrome. Melorheostosis has been associated with a variety of conditions such as sclerosing dysplasia and overlap syndrome (co-occurrence of melorheostosis with osteopokilosis and osteapathia striata) [4,5]. Anomalies of the blood vessels and lymph vessels were also reported [6,7]. Other associations include tumors: osteosarcomas, malignant fibrous histiocytoma, intrathecal lipoma, and desmoid tumors [8-11]. Minimal change nephrotic syndrome, phakomatoses, mesenteric fibromatosis, capillary hemangioma, and hypophosphataemic rickets have also been reported [7,12,13]. No pediatric cases of melorheostosis have been associated with carpal tunnel syndrome. We would therefore like to report an interesting case showing this association in a pediatric patient.

## Case presentation

Our case is that of an 8-year-old girl with a known condition of melorheostosis of the upper limbs. She presented a history of surgery for trigger fingers in both thumbs as well as the fourth finger of the left hand with follow-up consultations showing improvement in mobility and stiffness.

**Figure 1:**
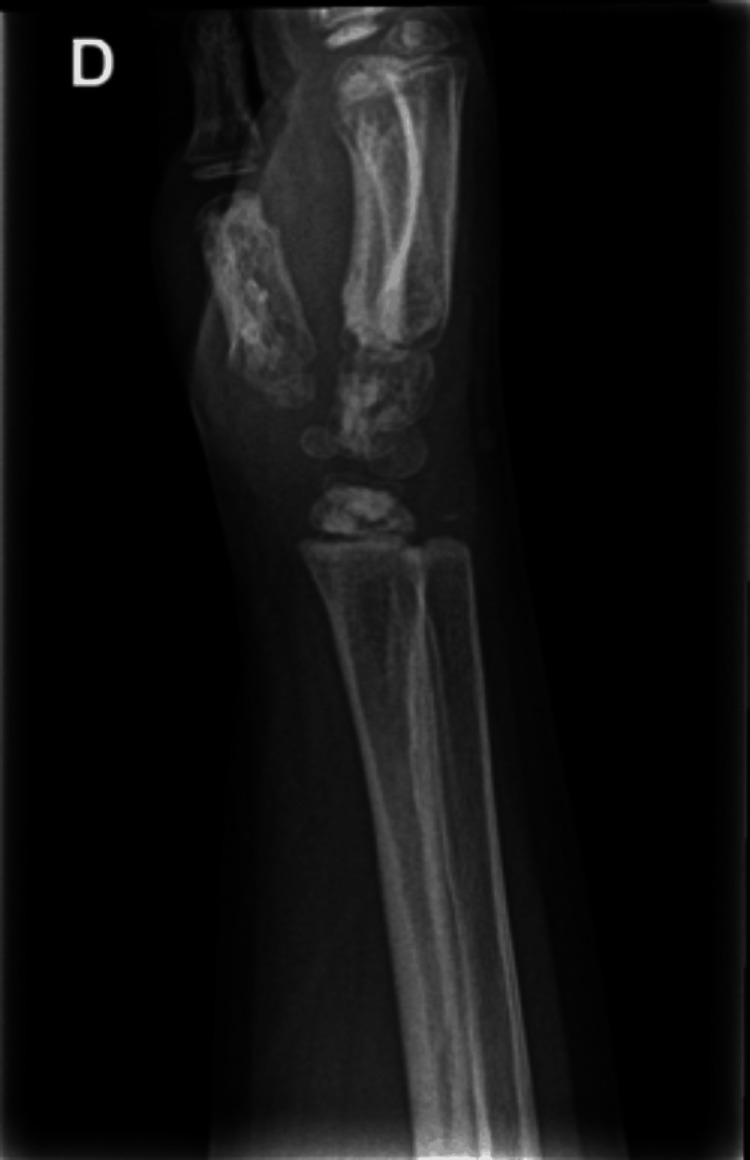
Plain radiography (profile) of the right wrist. Hyperdensities within the carpal bones.

**Figure 2:**
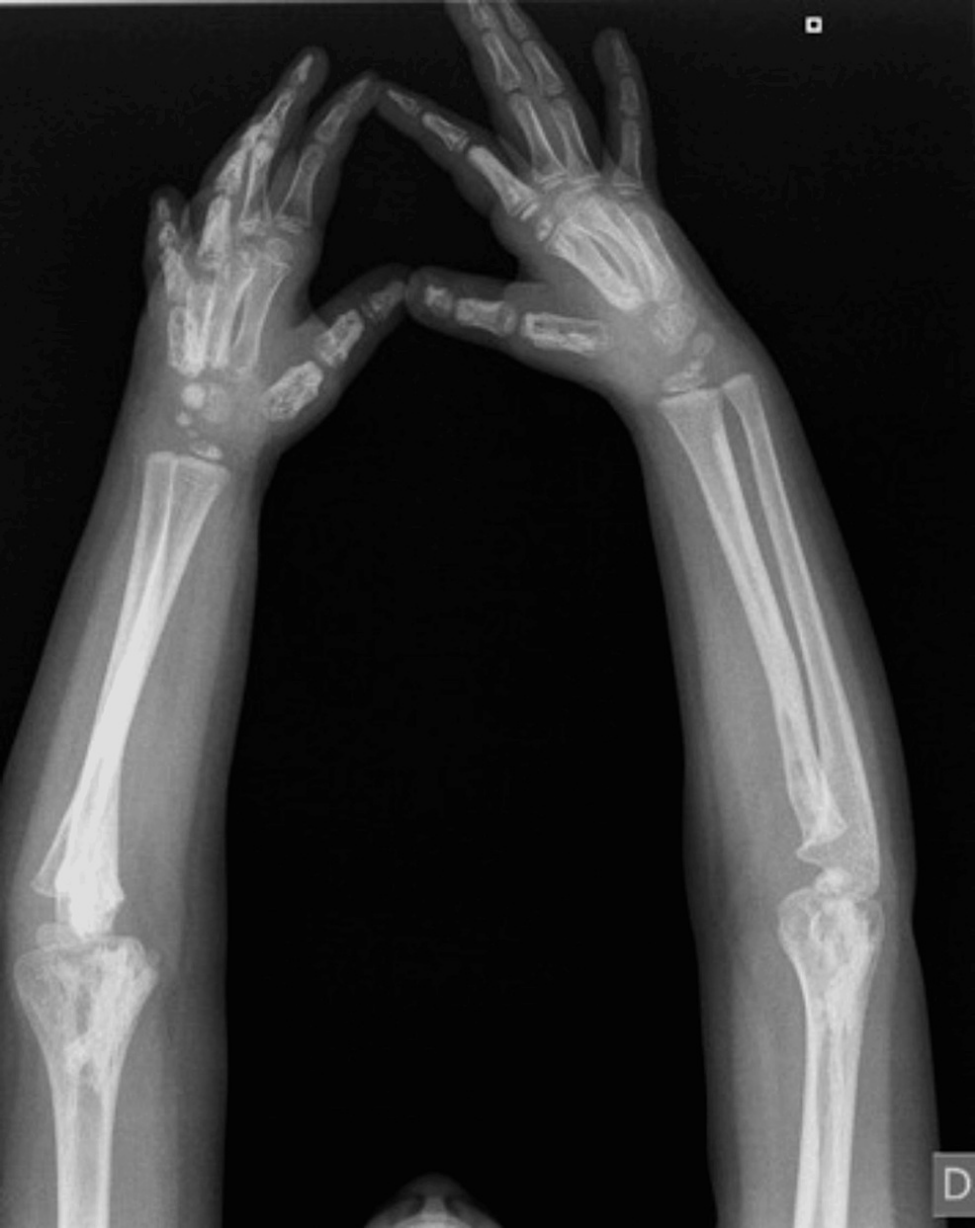
Plain radiography of both forearms and hands. Cortical hyperostosis of the radial and ulnar bones.

**Figure 3:**
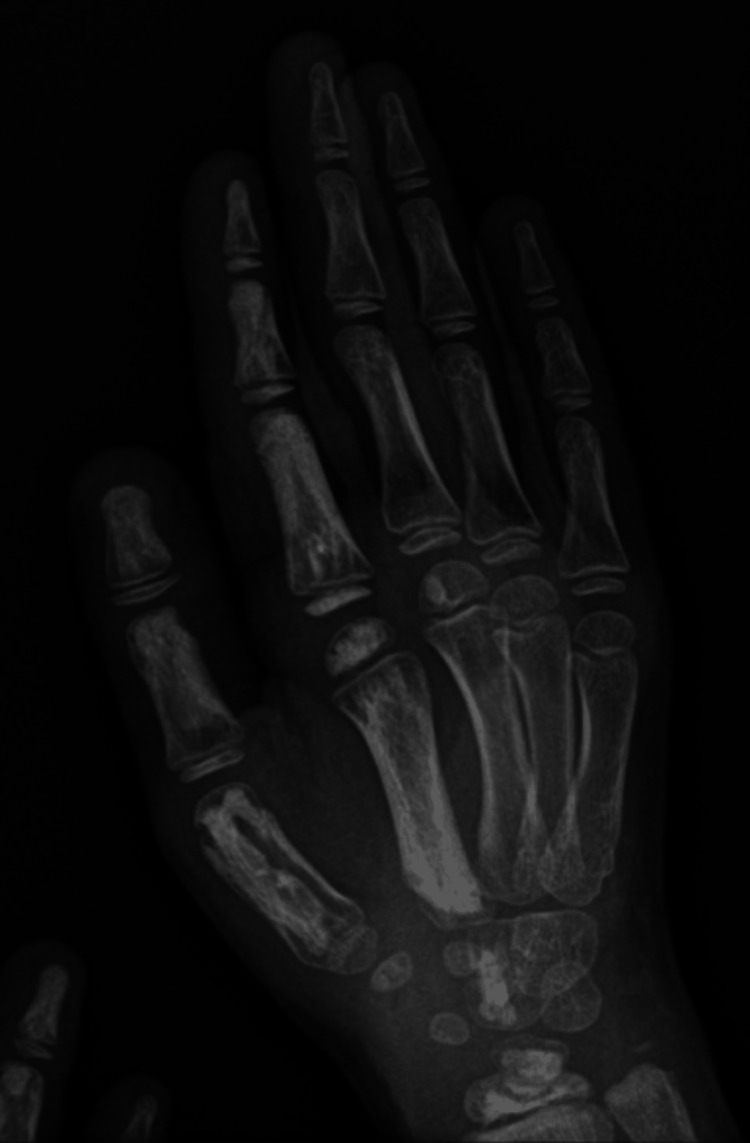
Plain radiography of the right hand. Hyperostosis and cortical fenestration of the wrist and carpal bones of the right hand.

The diagnosis was made at the age of 4 years when her mother noticed limitations in wrist extension. Wrist and elbow radiographs were performed at the time. These showed hyperdensities within the carpal bones, metacarpal bones, and cortical hyperostosis of the radial and ulnar bones (Figures 1, 2), wrist and marked bone hyperostosis and cortical fenestration of the wrist and carpal bones of the hand (Figure 3).

**Figure 4:**
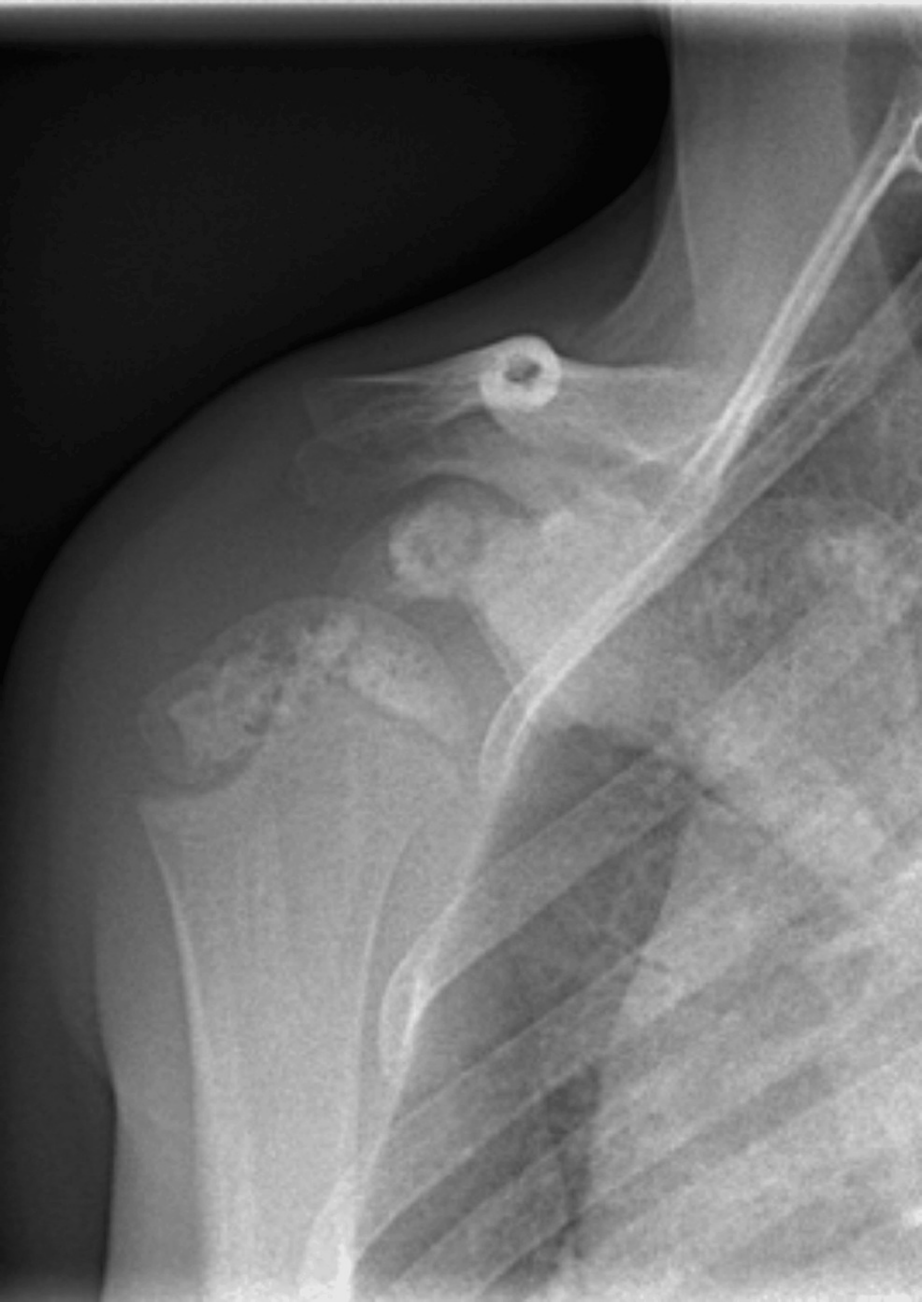
Plain radiography of the right shoulder. Hyperostotic lesions in the epiphyseal region of the right humeral head.

Follow-up radiographs were performed a year later as the patient developed progressive stiffness in the entirety of the upper limbs. These showed hyperostotic lesions in the humeral head (Figure 4).

Since then she developed paresthesia in both hands and a reduction in finger strength particularly in the first three fingers. Electromyography was normal.

Visually, we noted deformities in both hands with a flexed resting position, with no stiffness or pain. She was referred to our consultation for an ultrasound of both hands.

**Figure 5:**
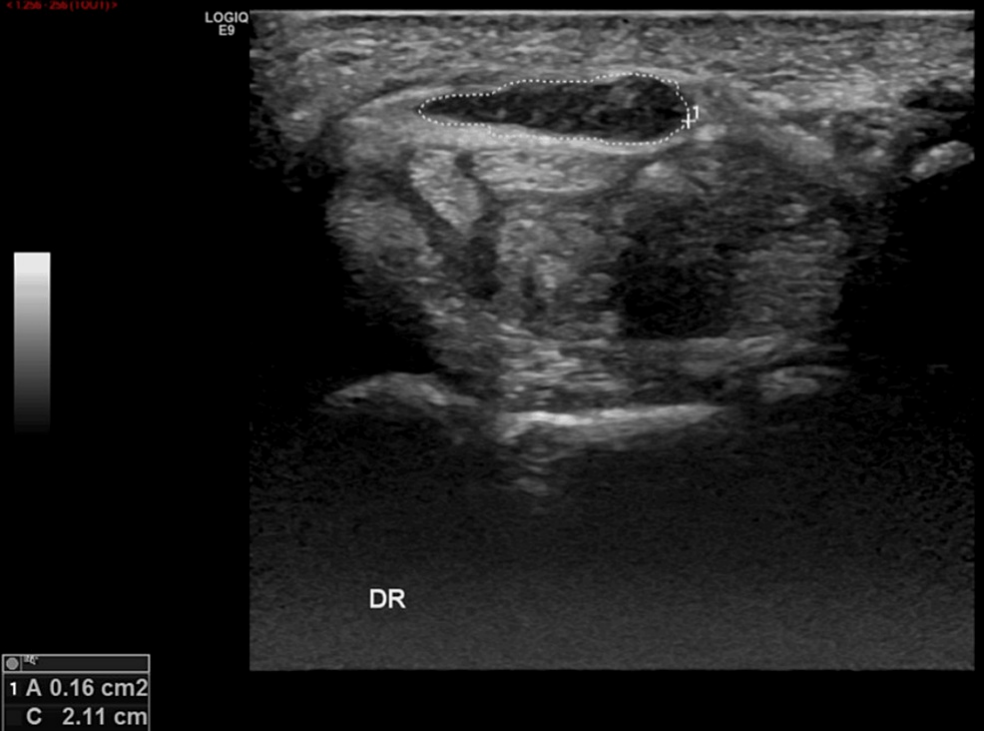
Ultrasound of the right median nerve. Thickening (16mm2) of the right median nerve proximally to the carpal tunnel.

We noted a thickening (16mm2) of the right median nerve proximally to the carpal tunnel (Figure 5) with a restoration of the normal caliber more distally.

**Figure 6:**
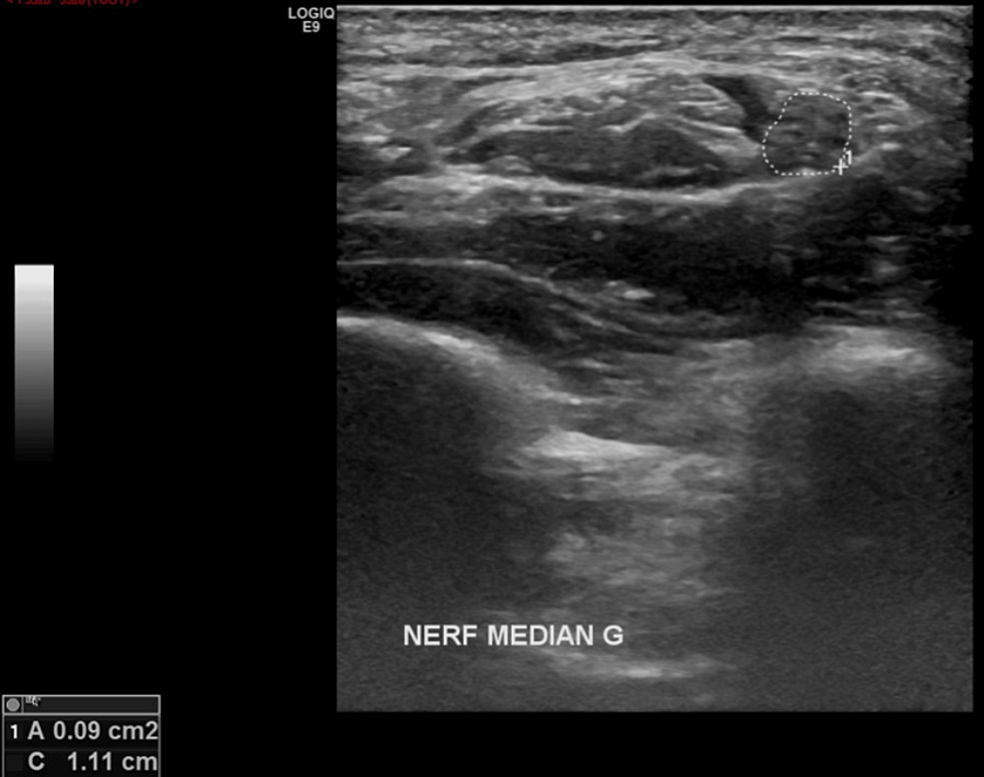
Ultrasound of the left median nerve. The left median nerve showed a thickening (9mm2) in the distal portion of the forearm with significant morphological alterations: flattening of the nerve hardly differentiated from the adjacent flexor retinaculum.

The left median nerve also showed a thickened (9mm2) and flattened aspect (Figure 6). Dynamic maneuvers showed a clear reduction of mobility of the nerve. Moderate atrophy of the thenar muscles was also found.

The right ulnar and radial nerves as well as the tendons showed no abnormalities.

More distally within the carpal tunnel, the nerve was not easily identified. Dynamic maneuvers showed an absence of nerve mobility which seemed to be practically incorporated with the adjacent and thickened flexor retinaculum. The median nerve branches were thickened distally. The ulnar nerve within the cubital tunnel was also thickened (13mm2). Flexor muscle tendons (especially of the fourth finger) and their sheaths were also thick, hypoechoic, and showed no vascularization. Important atrophy and adipose infiltration were noted within the left thenar muscles.

The left radial nerve showed no abnormalities.

## Discussion

Melorheostosis has a chronic course with episodes of exacerbation and arrest [14]. Clinical presentation can be varied but includes mainly pain, deformities stiffness, and joint movement restriction (due to contracture and fibrosis). The patient may develop limb shortening or lengthening and angular deformity. The patient may also remain perfectly asymptomatic [15]. In some cases, it may result in muscle atrophy as described in two cases by Judkiewicz et al. [16]. The first case was due to an impingement of the supra-scapular nerve (from scapular melorheostosis) and the second case was due to soft-tissue mass with sciatic nerve compression. In our patient the etiological factor is unclear but the gradual deformation of the wrist may have caused the narrowing of the carpal tunnel and by extension a median nerve impingement inducing a carpal tunnel syndrome with subsequent muscle atrophy of the thenar muscles.

Very few cases have described an association of melorheostosis with carpal tunnel syndrome although melorheostosis is commonly associated with soft tissue abnormalities of the affected limb (as mentioned above). One case by Bostman et al. reported the presence of severe carpal tunnel syndrome in a middle-aged woman with melorheostosis of the right upper limb since childhood [17]. One reported case of a 20-year-old woman with right-hand deformity and numbness was found to have a thickened flexor retinaculum and degenerated median nerve after surgery for carpal tunnel release [18]. Another report emphasized the importance of considering melorheostosis in pediatric patients presenting with soft tissue swelling joint contractures and sequelae such as carpal tunnel syndrome or trigger finger [19]. No other articles or studies were found associating the two conditions.

## Conclusions

To conclude, melorheostosis, although rare and described to be a benign condition, can be extremely debilitating for many known reasons (deformity, limb stiffness and pain). The lesser known association with neuropathic abnormalities must be kept in mind because of the dramatic consequences of such pathologies (irreversible destruction of the nerve) when they impact important nerves such as the median nerve. With this case, we would like to bring to light this possible association, and even more so as it relates to a pediatric patient where the median nerve has been dramatically altered and possibly irreversibly damaged.
